# Plexiform neurofibroma in the ear canal of a patient with Type I Neurofibromatosis

**DOI:** 10.1016/S1808-8694(15)30849-1

**Published:** 2015-10-18

**Authors:** Mauro Geller, Luiz Guilherme Darrigo Junior, Aguinaldo Bonalumi Filho, Marcia Goncalves Ribeiro

**Affiliations:** 1 Full professor of immunology and microbiology, Faculdade de Medicina da FESO. Coordinator of the Genodermatosis Sector, Clinical Genetics Unit, IPPMG-UFRJ. Member of the Board of Directors of the International Neurofibromatosis Association. Graduation course in neurogenetics, Harvard University - Massachusetts General Hospital.; 2 Resident in pediatrics, Hospital do Servidor Público Municipal de Sao Paulo. Specializing in clinical pediatrics, Universidade de Sao Paulo - USP.; 3 Graduate professor of dermatology, Hospital Naval Marcílio Dias. Research Fellowship, Harvard Medical School, Massachusetts General Hospital.; 4 Adjunct professor of clinical genetics, Faculdade de Medicina da Universidade Federal do Rio de Janeiro, UFRJ. Head of the Clinical Genetics Unit, IPPMG, UFRJ. Specialist in clinical genetics.; Study undertaken at the Centro Nacional de Neurofibromatose (CNNF). Reference Units: Instituto de Dermatologia Professor Rubem David Azulay da Santa Casa de Misericórdia do Rio de Janeiro, Instituto de Pediatria e Puericultura Martagão Gesteira da Universidade Federal do Rio de Janeiro, and Departamento de Imunologia e Microbiologia da Faculdade de Medicina de Teresópolis.

**Keywords:** plexiform neurofibroma, neurofibromatosis type 1

## INTRODUCTION

Type 1 neurofibromatosis (NF-1) is a multisystem genetic disease with significant cutaneous manifestations such as café-au-lait spots, freckles and neurofibromas.^1^ The incidence of NF-1 is about 1 : 2 500 new births; it affects equally all races and both sexes.^2^ Estimates show that there are currently in Brazil about 80 000 cases; worldwide, there are about 1.5 million cases of NF-1.^3^

A diagnosis of NF-1, according to criteria established by the “National Institutes of Health” (NIH) in 1987 and updated in 1990, depend on a careful clinical examination of the patient, his or her parents and siblings, and a detailed family medical history; at times, laboratory exams are needed.^3^ The plexiform neurofibroma (PN), also named plexiform neuroma, pachydermatocele or neurofibromatous elephantiasis, has been classified as a benign tumor of peripheral nerve sheath involving multiple nerve fascicles.^3^ It is a highly vascularized, slow-growing and locally invasive non-metastatic tumor.^3^ PNs are one of the important complications of NF-1; it may occur in infancy and rarely after adolescence. Although frequent in patients with NF-1, PNs are not pathognomonic of NF-1.^4^ The most common site is the trunk (43%), followed by the head and neck (42%) and limbs (15%).^5^ PNs may give rise to malignant peripheral nerve sheath tumors (MPNST) that in the past were referred to as neurofibrosarcomas or malignant schwannomas; these are the main cause of death and the most common malignancies in this group of patients.^3^

NF-2 is characterized by bilateral vestibular schwannomas, rarely with cutaneous manifestations. Typical lesions are schwannomas that may be present in the acoustic nerve or in other cranial nerves (nerve V, and sometimes nerve X) and meningiomas.^3^

## CASE STUDY

**Figure 1:**
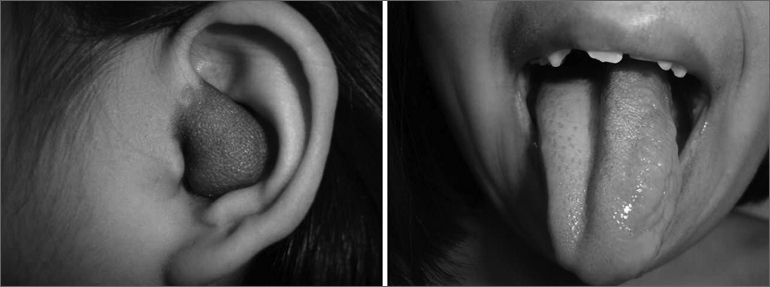
Plexiform neurofibroma in the left auditory canal and the tongue.

KJN, a female Caucasian patient aged 8 years and 4 months, from Peru, had NF-1 diagnosed at age 7 years; it was the first case in her family. Currently she has randomly distributed café-au-lait spots, and bilateral axillary and inguinal and thoracic freckles. The patient has a PN in the left auditory canal (Fig. 1), on the face (angle of the mandible, submandibular area and left temporal area), and on the left portion of the tongue (Fig. 1). Other findings included mild diffuse hyperpigmentation on the back of both hands, bilateral bone calluses on the back of the feet. Lisch nodules (iris hamartomas) were absent. Audiometry revealed severe conductive hearing loss in the left ear. Magnetic resonance imaging revealed anomalous impregnation by the paramagnetic contrast agent. Areas of signal changes, serpiginous lesions, and macroglossia were also found. There was a tumor on the left external auditory canal. This syndromic clinical picture suggested a diagnosis of PN.

## DISCUSSION

PNs are an important cause of clinical complications in NF-1; they develop mainly in childhood and adolescence. The natural history of PNs may vary significantly; some lesions may be quiescent for long period, while others may grow aggressively, especially during childhood and adolescence.^2^ PNs require clinical monitoring; tumor growth may cause pain, which may also suggest malignant transformation, 2 which occurs in 2 to 5% of PN patients.^2^

PNs are relatively common manifestations in NF-1 patients, and may increase their morbidity and mortality.^2^ According to Marocchio LS et al., PNs in the mouth of NF-1 patients is uncommon; few such cases have been described in the literature.^6^

## FINAL COMMENTS

We describe a case of NF-1 with a PN in the left auditory canal, resulting in severe ipsilateral hearing loss. This is an uncommon site in NF-1 patients. In contrast, auditory tumors are commonly found in NF-2 patients. To our knowledge, this is the first description of a case of PN in the auditory canal in an NF-1 patient.
